# Identification of Bacteria in the Sputum of a Cystic Fibrosis patient; A Comparison of Phenotypic and Molecular Methods

**DOI:** 10.2174/1874285801711010384

**Published:** 2017-12-29

**Authors:** Mubarak Alfaresi, Bassam Mahboub

**Affiliations:** College of Medicine, University of Sharjah, UAE

## Abstract

**Background:**

Cystic fibrosis (CF), caused by mutations in the CF transmembrane conductance regulator gene, is a common autosomal recessive disease. Accurate isolation and identification of the bacteria underlying these infections are is critical to the therapeutic management of CF.

**Objective:**

To compare phenotypic bacterial identification with a molecular method in a CF patient sputum.

**Methods:**

Bacterial identification done by standard microbiological method from a CF patient. Same sample underwent a molecular method involving 16S rDNA amplification, cloning, and sequencing.

**Results:**

All isolated bacteria from culture were also found after cloning PCR Product. Conversely, 9 pathogenic bacterial species were only detected after PCR and cloning.

**Conclusion:**

This study supports prior suggestions that a sequence-based molecular approach to clinical microbiology can significantly enhance the standard clinical culture-based view.

## 1 INTRODUCTION

Cystic fibrosis (CF), caused by mutations in the CF transmembrane conductance regulator gene, is a common autosomal recessive disease. The chronic bronchopulmonary infections that occur in CF patients decrease lung function and are the primary cause of the high morbidity and mortality associated with this disorder [1].

Accurate isolation and identification of the bacteria underlying these infections are critical to the therapeutic management of CF [2]. The present study compared the phenotypic identification of bacteria in the sputum of a 13-month-old CF patient to a molecular method involving 16S rDNA amplification, cloning, and sequencing.

## 2 MATERIAL AND METHODS

Agar plates of various media were inoculated with the CF sputum and colonies were grown. Standard microbiological methods and procedures were followed; full details of the methods are available upon request. Briefly, DNA was extracted, amplified using approximately 1,000 base pairs of the 16S rRNA gene through PCR and cloned. Finally, the purified PCR-amplified 16S rRNA inserts were sequenced and identified using the BLAST program (http:// www.ncbi.nlm.nih.gov/Blast.cgi), which compares the obtained sequence with those of the existing sequences in the GenBank database.

## 3 RESULTS AND DISCUSSION

Conventional phenotypic identification detected *Staphylococcus aureus, Pseudomonas aeruginosa*, and upper respiratory tract microbiota. Using the genomic method, 14 different bacterial species were identified (Table **1**). All isolated bacteria from culture were also found after cloning PCR Product. Conversely, 9 pathogenic bacterial species were only detected after PCR and cloning, including 7 *Streptococcus mitis*, 4 *Streptococcus parasanguinis*, 4 *Veillonella* species, 3 *Bacteroidetes species*, 2 *Prevotella* species, 2 *Neisseria macacae*, 1 *Streptococcus sanguinis*, 1 *Streptococcus pneumoniae*, and 1 *Granulicatella adiacens*.

Of importance, 81% of the isolated bacteria were only identified after cloning and sequencing. Genomic analysis of sputum yielded 49 sequences not recovered from culture and identified 9 different bacterial species.

## CONCLUSION

This study supports prior suggestions that a sequence-based molecular approach to clinical microbiology can significantly enhance the standard clinical culture-based view [2-4].

## Figures and Tables

**Table 1:** Bacteria detected using the genomic method

**Aerobic species**	**No. of clones**
*Pseudomonas aeruginosa*	20
*Neisseria macacae*	2
*Streptococcus mitis*	7
*Streptococcus sanguinis*	1
*Staphylococcus aureus*	1
*Streptococcus parasanguinis*	4
*Streptococcus pneumoniae*	1
*Granulicatella adiacens*	1
***Anaerobic species***	**No. of clone**
*Prevotella spp.*	2
*Veillonella parvula*	2
*Bacteroidetes sp.*	3
*Veillonella sp.*	2
*Veillonella dispar*	1
*Veillonella ratti*	1
